# Caloric Restriction and Its Effect on Blood Pressure, Heart Rate Variability and Arterial Stiffness and Dilatation: A Review of the Evidence

**DOI:** 10.3390/ijms19030751

**Published:** 2018-03-07

**Authors:** Rachel Nicoll, Michael Y. Henein

**Affiliations:** Department of Public Health and Clinical Medicine and Heart Centre, Umea University, 901 87 Umea, Sweden; michael.henein@medicin.umu.se

**Keywords:** blood pressure, heart rate variability, arterial stiffness, flow-mediated dilatation, caloric restriction fasting

## Abstract

Essential hypertension, fast heart rate, low heart rate variability, sympathetic nervous system dominance over parasympathetic, arterial stiffness, endothelial dysfunction and poor flow-mediated arterial dilatation are all associated with cardiovascular mortality and morbidity. This review of randomised controlled trials and other studies demonstrates that caloric restriction (CR) is capable of significantly improving all these parameters, normalising blood pressure (BP) and allowing patients to discontinue antihypertensive medication, while never becoming hypotensive. CR appears to be effective regardless of age, gender, ethnicity, weight, body mass index (BMI) or a diagnosis of metabolic syndrome or type 2 diabetes, but the greatest benefit is usually observed in the sickest subjects and BP may continue to improve during the refeeding period. Exercise enhances the effects of CR only in hypertensive subjects. There is as yet no consensus on the mechanism of effect of CR and it may be multifactorial. Several studies have suggested that improvement in BP is related to improvement in insulin sensitivity, as well as increased nitric oxide production through improved endothelial function. In addition, CR is known to induce SIRT1, a nutrient sensor, which is linked to a number of beneficial effects in the body.

## 1. Introduction

Primary, or essential, hypertension comprises around 90–95% of all cases of hypertension and is defined as high blood pressure due to genetic, environmental or lifestyle factors, such as obesity, excess salt, alcohol, lack of exercise and smoking [1,2]. Blood pressure (BP) is known generally to increase with age in the developed world [3] and is associated with metabolic abnormalities such as insulin resistance and hyperlipidaemia [4]. Elevated BP is associated with an increased risk for ischaemic heart disease, heart failure, stroke, peripheral artery disease, chronic kidney disease, cognitive impairment and cardiovascular mortality [5,6]. 

Pulse pressure, or heart rate, is often measured with BP. An elevated resting heart rate is associated with elevated BP through the baroreceptor reflex and is also independently associated with increased risk of all-cause mortality and cardiovascular (CV) complications [7]. The baroreceptor reflex is a homeostatic mechanism which helps to maintain blood pressure at near constant levels by means of a rapid negative feedback loop. Decreased heart rate variability (HRV), an indicator of disturbed cardiac autonomic function, may also be a predictor of all-cause mortality and CV comorbidities in the general population [7,8] and particularly in type 2 diabetes [9,10]. Dominance of the sympathetic nervous system (SNS) over the parasympathetic nervous system (PNS) will accelerate the heart rate and raise blood pressure, and itself appears to be a strong predictor of CV disease [11].

Also closely linked with BP, is arterial stiffness, usually measured by pulse wave velocity (PWV), although PWV may be an important predictor of CV morbidity and mortality, independently of BP [12]. Additionally, elevated BP is preceded by impaired endothelial function [3], which is known to lead to development of atherosclerosis and acute CV syndromes, likely through impaired nitric oxide synthesis [13,14]. Flow-mediated dilatation (FMD) is commonly used to assess endothelial function and is associated with most coronary heart disease risk factors [15], although it may also be predictive of CV events though endothelium-independent mechanisms [14].

While antihypertensive medications are effective in lowering BP, they may be expensive and have adverse effects that impair quality of life and reduce adherence [16]. There is also limited evidence that they have any beneficial impact on heart rate and its variability, PWV or FMD. The seventh report of the Joint National Committee on Prevention, Detection, Evaluation, and Treatment of High Blood Pressure (JNC-7) [17] recommends that the initial treatment strategy for lowering BP should be lifestyle modifications. But which lifestyle modification is preferable? There have been many studies of different types of diet, with the Dietary Approaches to Stop Hypertension (DASH) proving among the more successful [18,19]. Others have focused on diets for weight loss, since this in itself is associated with decreased CV morbidity and mortality, while it is well-established that reduction of weight or fat mass can generally lower BP [6,20]. 

There have been many studies showing that fasting or some form of caloric restriction (CR) has considerable success in lowering blood pressure. Many of these studies assume that this success is due purely to the weight loss, while others hypothesise that it is due to the lowering of insulin resistance. This review examines the studies of fasting or CR from 1990 onwards which are written in English, to determine how effective they are in improving BP, heart rate, heart rate variability and autonomic function, PWV, endothelial function and FMD, and whether there is a unifying mechanism of effect. Studies of religious fasting have been excluded. 

## 2. Caloric Restriction: Effects on Lowering Systolic and Diastolic Blood Pressure

Body weight and obesity, specifically visceral adiposity, are known to be independent risk factors for hypertension [6], and the lowering of these risk factors by CR can generally reduce BP. Both randomised controlled trials [21,22] and non-randomised studies [20,23,24,25,26] of CR have shown that it can lower systolic blood pressure (SBP), diastolic blood pressure (DBP) and mean arterial pressure in male and female type 2 diabetics and non-diabetics compared to a standard diet. Even studies as short as one or two weeks have found a significant improvement [26,27], with those of normal weight or mildly overweight also being seen to benefit [28]. In a study measuring BP at different times during the working day, intermittent fasting produced a significant decrease in office and ambulatory BP, but there was no difference in BP at home [29]. Studies of medically supervised fasting and CR tend to be much shorter and generally show beneficial results, independent of sodium intake [30,31,32,33]. In two such water fasting studies, approximately 82% of the borderline hypertensives achieved BP ≤ 120/80 mmHg [34], and among hypertensives almost 90% ceased to be hypertensive after 10-11 days and were able to discontinue all antihypertensive medication [35]; the greatest BP decrease was observed in subjects with the highest baseline BP. In many studies, BP continues to fall for up to 12 months after the end of the study. The recent MONET study [36], however, showed no change in BP after six months’ CR; the authors do not attempt an explanation for the failure to improve BP but it may be because the diet used for CR was the standardised diet recommended by American Heart Association (AHA) (low fat, high carbohydrate), whereas numerous studies have shown that metabolic health is improved with a low carbohydrate, higher fat diet [37,38]. 

Intermittent or alternate day fasting (ADF) produced mixed results [39,40,41], but this may be due to the fact that many of the subjects were normotensive at baseline [42,43,44], suggesting that the hypertensive status of the participants may be the principal determinant of BP improvement. This lack of result in normotensives in fact provides reassurance that fasting will not induce hypotension, with risk of dizziness and falls. A head-to-head comparison of continuous CR and ADF showed equal success with both methods, although a randomised controlled trial (RCT) found no success with either method [45], but this may again be because the subjects were normotensive at baseline. 

There have also been several cross-sectional studies investigating individuals who have practised CR over 3–15 years, compared to non-fasters. Without exception, the group practising CR had significantly lower blood pressure [8,46,47], with both SBP and DBP falling into the range found in 10-year olds [48]. Long term fasters tend to avoid refined foods, salt and trans fatty acids, they eat nutrient-dense unprocessed foods that supply >100% of the recommended daily intake for all essential nutrients, and they consume an energy intake which is 30% lower than those on the normal Western diet [8]. 

In the CR phase, certain types of foods are more successful than others. Liquid intake is more effective in reducing weight than solid food, but in an intermittent CR study by Klempel et al. [49], SBP and DBP were not altered by either intervention. The authors mention the accumulating evidence suggesting that a 5% reduction in body weight is required to decrease BP, which did not occur in this case, probably because the subjects reduced calories for only one day per week for eight weeks, which is not a particularly strict regimen; furthermore, baseline BP was already optimal. Several studies address the low fat versus low carbohydrate controversy. Meckling et al. [50] investigated the effects of continuous CR with a carbohydrate to protein ratio of 3:1 versus 1:1 versus control for 12 weeks; all participants were normotensive or pre-hypertensive. Although all groups decreased BP, the lower carbohydrate diet proved to be superior to both the control diet and the high-carbohydrate diet. Other studies have found no difference between the two diets, but these studies were carried out in subjects who were already normotensive [15,51]. A couple of studies of six months’ duration have used the American Heart Association (AHA) guidelines for the subjects’ diet (30% of energy as fat, 55% as carbohydrate and 15% as protein) but neither showed any improvement or difference between the intervention and control groups [37,52]. 

A number of studies have investigated the effects of CR versus exercise or the combination for lowering blood pressure. Among subjects who were not hypertensive, there was in general no added benefit of exercise for BP reduction over CR alone [24,50,53,54,55,56], although the addition of exercise may sometimes increase the reduction in DBP [57]. An RCT by Blumenthal et al. [58] found that in mostly hypertensive subjects, the addition of exercise reduced BP by mean 16.1/9.9 mmHg over four months, providing a degree of BP-lowering which exceeds that seen with a high dose of an antihypertensive drug [59]. At the end of treatment, a higher percentage were still hypertensive on CR alone relative to CR + exercise group, suggesting that in hypertensives, the effects of CR and exercise may be additive. 

Table 1 shows the major studies investigating fasting or CR and lowering of blood pressure with a subject population of >50. This demonstrates that virtually all show a beneficial effect on blood pressure, regardless of whether the subjects are normal weight, overweight, obese, have T2D or metabolic syndrome or are hypertensive or pre-hypertensive, or whether water fasting, a very low-calorie diet or 25% CR; only a low fat, high carbohydrate CR appears to have no beneficial effect. Similarly, the study duration varied in successful studies from 10–11 days to six months. 

### 2.1. Multivariate Regression Correlates with Blood Pressure Lowering

Studies showing a significant reduction in BP through CR are divided over which metabolic component correlates most accurately with the improvement in multivariate regression analysis. Curiously, no study shows that weight loss correlates with BP reduction [25,41,42,43], except for two very short-term studies which were carried out under medical supervision [30,31]. As well as being shorter, these supervised studies may also be more carefully controlled with no opportunity for cheating. Yet there remains one medically supervised study which shows no correlation between BP reduction and weight loss, but this study is a combination of a short supervised weight loss programme and a longer follow-up period, during which weight was regained but BP continued to fall [34]. This would suggest that the reduction in BP during the first two weeks may indeed correlate with weight loss, but non-supervised weight loss studies only look at the end point which tends to be at least eight weeks after baseline. BP reduction was similarly not correlated with insulin levels or insulin resistance [30,42]. Instead, studies have variously found a correlation between BP improvement and age [30], BMI [24], visceral fat [54] and waist circumference [55], although one found no correlation with BMI and body fat [41]. 

### 2.2. Does a Baseline Diagnosis of Metabolic Syndrome Influence the Effectiveness of CR?

Since elevated BP is a component of metabolic syndrome, it might be helpful to see how metabolic syndrome is affected by CR or fasting. The results of most CR studies appear to be unaffected by whether or not the subjects have metabolic syndrome [24,36,45], although a study of medically supervised weight loss found that subjects with baseline metabolic syndrome had a greater improvement in several parameters, including a BP reduction of 11/6 mmHg, despite BMI remaining >38 kg/m^2^; the number of subjects with metabolic syndrome also reduced significantly [31]. 

### 2.3. Does a Baseline Diagnosis of Hypertension or Pre-Hypertension Influence the Effectiveness of CR?

Studies of hypertensives are usually highly successful in lowering BP, even in studies lasting only two weeks or less [26,35,58,60]. In one of the short medically supervised water fasting studies by Goldhamer et al. [35] almost 90% achieved BP < 140/90 mmHg, with the average reduction being 37/13 mmHg; the greatest decrease was observed in subjects with stage 3 hypertension (>180/110 mmHg), who had an average reduction of 60/17 mmHg. Furthermore, during the supervised refeeding period (mean 6.8 days) there was a further 5.5/1.8 mmHg reduction in BP and 89% of baseline hypertensives became normotensive; all those who were taking antihypertensive medication at entry (6.3% of the total sample) successfully discontinued its use after fasting. 

Among those who are pre-hypertensive or normotensive, CR can also lower BP and pulse pressure [20,34,50]. After two weeks of medically supervised water fasting, 82% of pre-hypertensive subjects achieved BP ≤ 120/80 mmHg and a mean BP reduction of 20/7 mmHg, with the greatest decrease observed in subjects with the highest baseline BP [34]. However, it may be the case that in baseline normotensives the decrease in BP is not significant, providing reassurance that CR and fasting will not induce hypotension [15,42,51]. Whereas exercise proved useful in lowering BP among hypertensives [58], in general there was no added benefit of exercise over CR in non-hypertensives [24,50,53,54,55,56]. 

### 2.4. Does Ethnicity Influence the Effectiveness of CR in Blood Pressure Lowering?

Ethnicity appears to have little bearing on the BP response to CR. Because CV risk disproportionately affects black populations, Hong et al. [24] investigated the difference in effect of CR for 12 weeks in black and matched white females; both groups showed significant improvements in SBP and DBP, with no difference in response between ethnicities. Similarly, van Schinkel et al. [23] prescribed CR for male Caucasians and matched South Asians; South Asians have a higher risk of developing cardiovascular disease (CVD) compared to Caucasians, and those with insulin resistance and T2D have a higher risk of cardiac complications. After eight days, SBP and DBP were significantly decreased in both groups with no difference between them. 

### 2.5. Is DBP Slower to Respond to CR Than SBP?

A few studies have found that whereas there is a significant reduction in SBP, DBP may be less responsive within the time-frame of the study. A four-week study of postmenopausal females [25] found a significant reduction in SBP, but although DBP initially declined, the changes were not consistently significant. The authors speculate that the relatively low weight loss (<5%) could account for the lack of response of DBP, although as reported above, few studies in this survey show a correlation of BP reduction with weight loss. Similarly, a two-week study of CR in obese hypertensive subjects found that SBP was significantly reduced over 24 h and in both daytime and night time, while DBP was reduced only in the daytime. 

Sasaki et al. [33] gave medically supervised CR in hypertensive subjects and normal controls and found that the reduction in SBP started with the beginning of CR, while DBP began to decrease after one week of CR, but both SBP and DBP reached a plateau a few days before the end of the two-week intervention. One study also found that DBP was less responsive, or possibly slower to respond, than SBP in subjects with metabolic syndrome. With Buchinger fasting [27], overall SBP and DBP decreased significantly, with SBP reducing by a mean 16.2 mmHg and DBP by 6.0 mmHg, but in subjects with baseline metabolic syndrome, the reduction in DBP was not significant, although their post-fast blood measurements were those of the baseline non-metabolic syndrome group, i.e., they had reversed their metabolic syndrome. Possibly a longer fast in this sub-group would have lowered DBP significantly. 

## 3. Caloric Restriction: Effects on Lowering of Heart Rate

Results for the effectiveness of CR in lowering heart rate are mixed. An RCT showed that heart rate was significantly lowered by CR in subjects with T2D [21], while among older long-term fasters (undergoing CR for between three and 15 years), 24-h, day time and night time heart rate was significantly lower compared to matched controls eating a normal diet and were similar to values found in healthy adults aged 20–30 years [8]. Nevertheless, a study of ADF showed that heart rate reduced in concert with SBP, but not with DBP, which was unchanged; reduction in heart rate was not associated with reduction in weight, BMI or percentage body fat [41]. The remainder of the studies measuring heart rate found no change with CR [15,46,51,52], even between tertiles of insulin resistance [40] or where BP fell significantly [23,60]. 

A liquid food CR was more effective at lowering heart rate than solid food, although BP was not altered by either intervention [49], while a meal timing ADF study showed a decrease in heart rate only in those who consumed all their calories at lunch, as opposed to dinner or three isocaloric small meals throughout the day [42]. The addition of exercise to CR also shows mixed results, with an RCT finding no benefit to resting heart rate [61], although a six-month study showed that CR + exercise induced a reduction in heart rate, which was not achieved by CR alone [53], while maximal heart rate decreased with exercise over 12 weeks, regardless of diet [50]. 

## 4. Caloric Restriction: Effects on Heart Rate Variability and Autonomic Nervous System Balance

CR can generally significantly improve HRV and the balance between sympathetic and parasympathetic nervous system activity [53,62]. A study of long-term fasters showed that older fasters had HRV results which were similar to those found in a much younger cohort or were similar to results in hypertensive individuals taking atenolol, which reduces sympathetic and increases parasympathetic control of the heart [8]. Although Nakano et al. [60] failed to find a significant reduction in heart rate in a very small number of hypertensive subjects after two weeks, they did observe a significant rise of high frequency in night-time and fall of low frequency/high frequency in day-time. The authors suggested that in obese patients, the blunted night time rise in parasympathetic nervous function and fall in sympathetic nervous function may be a cause of high BP, but that CR may generate nocturnal sympatho-vagal balance improvement. 

Animal studies have also shown greater baroreceptor responsiveness to hypotensive stress with CR, although there was little response to hypertensive stress [63]. Decreased low-frequency power in DBP variability and increased high frequency power in HRV also indicate that CR leads to decreased sympathetic activity and increased parasympathetic activity, suggesting that long-term CR can slow the age-associated deterioration of autonomic nervous system function. Similarly, in male rats CR induced increases in the high-frequency component of HRV spectra, a marker for parasympathetic activity, and decreases in the low-frequency component of diastolic blood pressure variability spectra, a marker for sympathetic tone [64]. These parameters required at least 1 month to become maximal but returned toward baseline values rapidly once rats resumed ad libitum diets. 

Table 2 shows the studies investigating fasting or CR and lowering of heart rate and/or an improvement in heart rate variability, with a subject population of >20. This demonstrates the mixed results for heart rate, while the beneficial effects on HRV were seen in long-term fasters but the two-week study which showed no effect was too small to be included. 

## 5. Caloric Restriction: Effects on Pulse Wave Velocity

A 2015 systematic review and meta-analysis [65] of 20 weight loss studies found eight that had shown no effect on carotid-femoral or brachial-ankle PWV, as a measure of arterial stiffness, whereas 12 showed beneficial effects. A pooled analysis found that a mean weight loss of 8% was associated with a PWV reduction of 0.6 m/s, indicating significant clinical improvement; the BP reduction was a predictor of PWV improvement. Other studies have reflected these mixed results, with Blumenthal et al. showing that CR improved carotid-femoral PWV compared to a control group in subjects with elevated BP [58], although Weiss et al. showed no difference in carotid-femoral PWV or carotid augmentation index with either CR or exercise, despite a reduction in SBP with CR [61]; as the authors found a non-significant carotid-femoral PWV reduction of 0.6 m/s, they consider that the sample size possibly needs to be larger for the improvement to become significant. 

The study by van Schinkel et al. [23], suggests that degree of insulin resistance and ethnic differences may influence PWV. They found that in overweight South Asians and Caucasians, South Asians at baseline were more insulin resistant and had significantly higher PWV in the distal aorta, indicating a stiffer aorta. After a very low-calorie diet (VLCD) for eight days, aortic PWV was decreased only in Caucasians. The authors point out that insulin resistance and the presence of T2D compromise aortic elastic function, leading to increased wall thickness and arterial stiffening through trophic effects on smooth muscle cells. Since the South Asians were more insulin resistant at baseline than the Caucasians, this might help to explain why PWV was significantly decreased only in Caucasians. In animals, PWV was 27% higher in aged mice compared to young mice, although lifelong CR prevented the age-associated increase [66]. 

## 6. Caloric Restriction: Effects on Endothelium-Dependent Flow-Mediated Dilatation

Two studies show a significant increase in FMD with CR, indicating improved endothelial function [20,67]; the benefits may be maintained for up to 12 months [67]. The study by Raitakari et al. [20] showed a baseline correlation of FMD with brachial artery diameter, waist-to-hip ratio and pack-years of cigarettes, with no difference between hypertensives and normotensives, but after CR, FMD increased by 60% regardless of gender, BP, smoking status or menopausal status, and there was similarly no association with brachial artery diameter or nitrate-mediated (non-endothelial) dilatation. The study by Khoo et al. also measured soluble E-selectin as an indicator of endothelial function and found that it too was significantly improved by CR [67]. A study investigating the effects of low fat CR vs. high fat CR in normotensive subjects found that although there was no effect on BP, yet the high fat group showed a significant decrease in brachial artery FMD while there was an increase in the low-fat group [15,51]. Nevertheless, another study of two low fat diets found that although SBP and PWV reduced significantly, FMD did not change [68]. In a 12-week study [69] of subjects with moderate hypertriglyceridaemia, brachial artery post-prandial peak FMD and weight loss were significantly increased in the carbohydrate-restricted CR group but decreased in the fat-restricted CR group, indicating that carbohydrate restriction improves postprandial vascular function compared to fat restriction. 

A study [33] of medically supervised CR for two weeks in obese Japanese subjects with essential hypertension and normotensive controls showed that baseline forearm blood flow was not different between the groups, despite a large difference in blood pressure. When given an infusion of acetylcholine, there was significantly attenuated blood flow in the hypertensive group compared to controls. Among the hypertensives, administration of CR was associated with significantly reduced BP, although forearm blood flow was no different. Again, CR increased the response of forearm blood flow to acetylcholine infusion, indicating improved endothelium-dependent vasodilatation; similarly, the change in forearm vascular resistance in response to acetylcholine significantly decreased. Intra-arterial infusion of *N*G-monomethyl-l-arginine (L-NMMA), a nitric oxide synthase inhibitor, significantly decreased the forearm blood flow response to acetylcholine, both before and after the intervention, indicating that the blood flow response to acetylcholine is mediated by nitric oxide. 

In the two studies assessing the effect of CR with or without exercise, neither intervention showed any effect on FMD, although one showed a significant decrease in BP and weight [55,57]. Wycherley et al. [55], who investigated diabetic subjects, also observed decreased oxidative stress and increased urinary nitrate/nitrite concentrations, suggesting increased NO production but they hypothesise that the lack of improvement in FMD indicates a disconnect between increased NO availability and the endothelium dilatory response, also found in another study of subjects with T2D [70]. Nevertheless, a long-term animal study [71] of diabetic rats allocated to CR, exercise or control, showed that thoracic aorta blood flow was significantly improved in the rats given exercise compared to the other two groups. [55]

Table 3 shows the studies investigating fasting or CR and lowering of blood pressure with a subject population of >20. This demonstrates that all show a beneficial effect on FMD, regardless of whether the subjects were overweight, obese, have T2D or are hypertensive. CR of 580–1500 kcal/day is an effective intake, although a low fat, high carbohydrate CR appears to have no beneficial effect. Similarly, the study duration varied in successful studies from two weeks to 12 weeks. 

### Multivariate Regression Correlates with Endothelium-Dependent Flow-Mediated Dilatation Improvement

Again, in multivariate regression analysis, weight loss appears not to be correlated with endothelium-dependent FMD improvement and there is also no correlation with changes in BP, body fat, insulin, insulin resistance or lipids [20,33,40,69], despite many studies showing that obesity is associated with decreased vasodilatation and endothelial dysfunction [20]. There may, however, be a correlation with blood glucose, although studies are divided. Raitakari et al. [20] showed that the strongest correlate with improvement in FMD is plasma glucose; the authors speculate that either the lack of correlation of FMD with weight change indicates a threshold effect, after which no further effect is seen, or that the provided CR diet contained substances that improved endothelial function, such as vitamins C and E and folic acid, but pointed out that these substances were included at the recommended daily allowance, whereas studies showing their beneficial effect on endothelial function had used much higher doses. Hoddy et al. [40] found the only correlate with improvement in FMD was adiponectin concentrations, which had increased in those with the lowest insulin resistance only. Although not studies solely of CR, others have found that insulin resistance is independently predictive of impaired FMD or peak increase in forearm blood flow [72,73]. 

## 7. Caloric Restriction: Effects on Endothelium-Independent Flow-Mediated Dilatation

Endothelium-independent FMD is normally measured by sodium nitroprusside or glyceryl trinitrate (GTN), which are believed to dilate blood vessels through production of nitric oxide, although the precise mechanism is disputed. The study by Wycherley et al. [55], described above, found no effect on endothelium-independent brachial artery FMD after CR or CR + aerobic exercise for 12 weeks. The authors note that previous studies had observed impaired dilatory response to the administration of GTN in subjects with type 2 diabetes compared with non-diabetics, suggesting that NO availability is not the limiting factor for FMD in T2D, but there may be structural changes within the arterial wall that dampen responsiveness to NO. A similar finding was reported by Sasaki et al. [33], with no response of forearm blood flow to isosorbide dinitrate infusion, either at baseline or after CR. 

The long-term study of diabetic rats [71], described above, had shown significant improvement in thoracic aorta blood flow after exercise only but there was no difference in blood flow between groups when sodium nitroprusside was administered, despite increased urinary excretion of nitrite in the exercise group. The authors suggest that exercise, but not CR, prevents endothelial dysfunction in diabetic rats, likely due to the exercise-induced increase in nitric oxide production.

## 8. Summary of Findings

CR is capable of significantly lowering SBP, DBP and mean arterial pressure in individuals with elevated BP, regardless of gender, ethnicity, BMI or a diagnosis of metabolic syndrome or T2D; nevertheless, DBP may sometimes be less responsive or slower to respond than SBP. BP can be normalised, even in studies as short as two weeks, and anti-hypertensive medication discontinued, and the BP-lowering may continue beyond the end of the CR period. Those with the highest BP show the greatest decrease, with some studies of normotensives showing no effect, which provides confidence that fasting patients are unlikely to suffer hypotension. The only studies which fail to show an effect have either been carried out in baseline normotensives or where a low fat, high carbohydrate diet was consumed during the CR period. Exercise conferred no added benefit unless the subjects were hypertensive, when it enhanced the CR effects. 

Heart rate may be significantly lowered by CR among type 2 diabetics and in long-term fasters, but in several studies, CR has no effect on heart rate, with or without exercise, even though there was a significant reduction in BP. Heart rate variability and the balance between the sympathetic and parasympathetic nervous system activity, however, are generally improved by CR. The results for the effects of CR on PWV are more equivocal, although a pooled analysis found that weight loss of 8% was associated with significant improvement, with BP reduction as an independent predictor. Unlike BP, ethnicity may have a bearing on results through a higher incidence of insulin resistance among South Asians relative to Caucasians. CR appears to generally increase FMD but may be more effective in hypertensives than normotensives; the additional improvement in E-selectin confirmed the benefit on endothelial function, although this may only be evident after an acetylcholine infusion. Although the improvement is likely mediated by nitric oxide, in type 2 diabetics increased availability of nitric oxide does not, in itself, guarantee improved FMD. The few studies investigating endothelium-independent dilatation have all shown no effect from CR. 

In multivariate regression, blood pressure reduction shows no correlation whatsoever with weight, insulin concentrations or insulin resistance, and no clear correlation with BMI, visceral fat or waist circumference. Even though obesity is associated with endothelial dysfunction and impaired dilatation, improvement similarly does not correlate with weight, body fat, insulin concentrations, insulin resistance, BP or lipids in multivariate regression. There may, however, be a correlation of FMD improvement with plasma glucose, again mediated through NO bioavailability and/or adiponectin, although this may depend on insulin sensitivity. The possibility of a threshold effect on BP and FMD with weight loss has been suggested in a couple of studies, but this has not been sufficiently investigated to determine whether it is time-or weight-loss dependent. 

## 9. Potential Mechanisms

**Figure 1:**
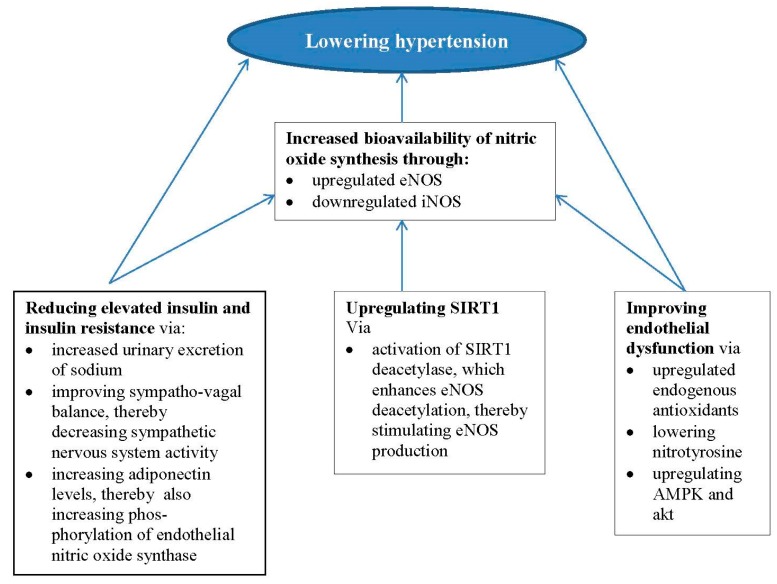
Potential mechanisms of the effects of CR on hypertension.

Although the precise mechanism(s) by which CR can lower BP and improve autonomic nervous system and endothelial function are as yet unclear, potential candidates include reducing insulin resistance, improving nitric oxide production and upregulating SIRT1. These potential mechanisms are summarised in Figure 1.

### 9.1. The Role of Insulin and Insulin Resistance

The relationship between hypertension and hyperinsulinaemia or insulin resistance is well known, with several studies showing that plasma insulin concentrations increase in obese hypertensives through resistance to insulin-mediated glucose disposal [33]. There is evidence that around 50% of patients with essential hypertension are also insulin resistant and it is this group which are more likely to develop CVD [74,75]. This association may be influenced by age; Jung et al. showed that in the most insulin-resistant tertile, the distribution of those hypertensive, pre-hypertensive and normotensive was approximately equal in those aged <52, while in those aged ≥52 the incidence of hypertension was five-fold higher compared to subjects with normal BP [76]. 

Hyperinsulinaemia can result in peripheral vasoconstriction, alteration in cation transport, enhanced renal tubule sodium reabsorption or elevation of the glomerular filtration fraction, vascular smooth muscle cell growth and hypertrophy and stimulation of the renin-angiotensin-aldosterone system [6,33,60]. It can also raise BP through stimulation of the sympathetic nervous system and downregulation of the baroreceptor reflex function, inducing high cardiac output and a sympatho-vagal balance which increased in direct proportion to the degree of hyperinsulinaemia, independent of BMI [60,77], although Nakano et al. note that sympathetic activation is known to induce insulin resistance and hyperinsulinaemia, which in turn stimulate further sympathetic nervous activity in a positive feedback loop [60]. By removing triggers for insulin secretion, thereby lowering insulin and insulin resistance, CR is interrupting that positive feedback loop. In addition, a study by Galvao et al. [72] showed an inverse correlation between homeostatic model assessment-insulin resistance (HOMA-IR) and FMD in obese non-diabetics, with those in the lowest tertile of insulin resistance having the highest FMD. 

Biochemistry studies have shown that in endothelial cells, hyperinsulinaemia and insulin resistance are associated with impairment of the inositol 3-kinase-dependent signalling pathway, which leads to the synthesis of nitric oxide, confirming that insulin resistance is associated with vascular dysfunction [69,72,78,79,80], while the increase in plasma asymmetric dimethylarginine (ADMA), an inhibitor of eNOS, correlates with elevated BP and insulin resistance [81,82]. This suggests that nitric oxide is at least one of the mediators of vasodilatation, as seen in a study showing a positive association between forearm endothelial NO synthesis and insulin sensitivity in healthy humans, whereas increased insulin resistance impaired endothelial function through disruption of NO production [33]. Others have noted that insulin may be linked to BP via microvascular dysfunction and that it demonstrates a vasoconstrictive effect via MAP kinase and the production of endothelin-1 [83]. Yet it has been observed that endothelial dysfunction itself can worsen insulin resistance by reducing blood flow, which appears to be caused by an imbalance between NO and endothelin-1 expression, suggesting another positive feedback loop [79].

The improvement in insulin sensitivity through CR has been associated with lower BP not only through increased NO synthesis, but also by increasing the urinary excretion of sodium and/or improving the sympatho-vagal balance [60,84]. In addition, obesity studies using catecholamine levels as a measure of sympathetic nervous activity have generally shown that the fall in BP with weight loss correlated with decreased plasma noradrenaline, although generally insulin levels and insulin resistance were not measured [85]. Similarly, a study by Ikeda et al. [86] found that BP improvement after weight loss in obese hypertensives was predicted by age and improvement in plasma noradrenaline, plasma renin activity and insulin sensitivity. In multivariate regression analysis, however, the only major independent predictor of the peak increase in forearm blood flow in obese adults after CR was HOMA-IR [73], suggesting that CR affects endothelium-dependent vasodilatation indirectly, through improving insulin sensitivity. In addition, the study by Hoddy et al. [40] found that adiponectin was inversely associated with insulin resistance, but CR was able to lower insulin resistance and raise adiponectin concentrations; adiponectin is known to stimulate the phosphorylation of endothelial nitric oxide synthase (eNOS), thereby increasing NO-dependent vasodilatation [87]. 

In an editorial, Anderson et al. [88] points out that hyperinsulinaemia has also long been associated with vasodilatation in skeletal muscle, the principle site of action of insulin, possibly through an efflux of intracellular calcium through the action of calcium ATPases. This would appear to contradict the findings that insulin resistance is associated with vasoconstriction and elevated blood pressure, but possibly the contradiction lies in the duration of elevated insulin concentrations and the baseline insulin sensitivity; short-term hyperinsulinaemia in insulin-sensitive individuals induces vasodilatation, while persistent hyperinsulinaemia can lead to insulin resistance, which impairs the vasodilatory response and induces a rise in blood pressure and reduced blood flow through the various mechanisms discussed above. A cross-sectional study by Zavaroni et al. showed that after an overnight fast, plasma insulin was the sole predictor of NO concentrations in essential hypertension, independent of age or measures of adiposity; plasma NO concentrations were significantly higher in the hypertensive group with insulin resistance [89]. Although there was no correlation between NO concentrations and BP, the authors consider that this result reflects insulin’s vasodilatory effect, which is dependent on endothelial NO production, and that the increased plasma NO concentrations represented a compensatory response to prevent increased BP in insulin resistant subjects. While this could indeed be the case, it is not the only study to show an absence of correlation between NO concentrations and BP, suggesting that one of the other insulin effects, such as activation of the sympathetic nervous system, had over-ridden the effect of NO. In fact, in vitro studies have shown that the production of endothelium-derived NO through the action of endothelial NO synthase (eNOS) is stimulated by increased shear stress [55], raising the possibility that hyperinsulinaemia is causing shear stress, either directly or indirectly. 

### 9.2. The Role of Endothelial Dysfunction

Impaired endothelial function precedes the development of prehypertension and hypertension [3], as seen in hypertensive rats which exhibited reduced vasodilatation to insulin prior to significant hypertension developing [9]. Chronic hyperglycaemia may be another mechanism that impairs endothelium-dependent vasodilatation, reducing NO bioavailability through decreased eNOS expression, inactivation of NO by glucose, activation of protein kinase C, formation of reactive oxygen species (ROS) and formation of advanced glycosylation end products (AGEs) [20]. Because this impairment of endothelial function can be reversed by pretreatment with antioxidants, the authors hypothesised that oxidative stress may be the underlying cause of the dysfunction [20]. 

Many studies show that improvement in BP and endothelial function is principally mediated by increased bioavailability of the vasodilator NO through increased activity of eNOS [90], but the improvement may be relative to the degree of oxidative stress rather than in absolute terms; superoxide anions are known to reduce endothelium-dependent vasodilatation by lowering production or release of NO [33]. Rats given short-term CR showed improved endothelial relaxation and a higher ratio of vascular NO levels relative to NADPH-sensitive superoxide production (O2^−^), reducing mRNA expression of NADPH oxidase (Nox) 1 and p22phox protein [91]. Ageing individuals suffer endothelial dysfunction as a result of the activation of inducible nitric oxide synthase (iNOS) rather than eNOS [92]; in ageing rats, impaired endothelium-dependent vasodilatation was associated with reduced eNOS and increased iNOS, oxidative stress was increased and endogenous antioxidants were downregulated, whereas CR restored endothelial function and reduced iNOS expression and oxidative stress [92]. Similarly, short-term CR in old mice induced the same level of endothelium-dependent dilatation as in non-CR young mice, restoring the reduced NO bioavailability, expression of eNOS and manganese superoxide dismutase and lowering nitrotyrosine, a cellular marker for oxidant modification, NADPH oxidase and superoxide production [93]. 

In the hypertensive rats above, which exhibited reduced vasodilatation, treatment with the PPARγ agonist, rosiglitazone, improved insulin sensitivity and significantly reduced BP through restoration of the activity of phosphatidylinositol-3 kinase (PI3K) [94]. CR may also modify the activity of the nutrient- and energy-sensing AMP-activated protein kinase (AMPK), along with akt, the survival kinase in the AMPK/PI3K/akt/eNOS signalling pathway [91,95,96]. A study using overexpression of adiponectin, rather than CR, induced a similar reduction in BP and activation of the AMPK/eNOS pathway, indicating that increased adiponectin may be a key mediator of the benefits of CR [96]. The other important adipokine, leptin, often elevated in the obese, may have the opposite effect by increasing sympathetic nervous system activity [33] and inhibiting the production of NO through the stimulation of ROS, which scavenge NO and impair eNOS function [97]. Other vasoactive substances may also be released, such as endothelium-derived relaxing factors and atrial natriuretic peptide, which are all reduced with age [30]. While increased NO bioavailability is regularly associated with a reduction in BP by CR, no significant correlation between reduction in BP and increased blood flow response to acetylcholine has been observed [33].

### 9.3. The Role of SIRT1

It appears that CR activates SIRT1 deacetylase, which enhances eNOS deacetylation through lysines in the eNOS calmodulin-binding domain, thereby stimulating endothelial NO production; inhibition of SIRT1 reduces NO bioavailability and inhibits endothelium-dependent vasodilatation [94,98]. An activator of SIRT1 normalised SIRT1 expression in aged mice and restored endothelium-dependent vasodilatation by enhancing cyclooxygenase (COX)-2 signalling and reducing inflammation and oxidative stress in the elderly animals [99]. Another animal study showed that CR-improved endothelial function was associated with restored eNOS phosphorylation and SIRT1 expression, but reduced eNOS acetylation [100]. In eNOS-deficient mice, CR failed to exert an antihypertensive effect, as evidenced by elevated BP and absence of SIRT1 activity [101]. 

## 10. Conclusions

CR is capable of significantly lowering SBP, DBP and mean arterial pressure, although DBP may sometimes be less responsive or slower to respond than SBP. BP can be normalised, even in studies as short as two weeks, and anti-hypertensive medication discontinued. Those with the highest BP show the greatest benefit, with some studies of normotensives showing no effect, which provides confidence that fasting patients are unlikely to suffer hypotension. Exercise conferred no added benefit unless the subjects were hypertensive, when it enhanced the CR effects. Heart rate and pulse wave velocity may not always improve with CR but heart rate variability and the balance between the sympathetic and parasympathetic nervous system activity are generally ameliorated. FMD is generally increased by CR, particularly in hypertensives, which may be related to improvement in endothelial function and nitric oxide production. CR has no effect on endothelium-independent dilatation. Multivariate regression shows no consistent independent correlate with either reduction in BP or improvement in FMD, although there may possibly be a threshold effect with weight loss. Potential mechanisms linking CR to improvement in BP and endothelial function comprise reduction in insulin resistance, improved nitric oxide production and induction of SIRT1. 

## Figures and Tables

**Table 1:** Summary of studies showing the effect of fasting or caloric restriction on blood pressure in >50 human subjects.

Study Author and Reference	Type of Study	Subjects	CR or No. of Calories	Duration of Study	Significant Lowering of
Raitakari et al. [20]	Obs	Obese	580 kcal/day	6 weeks	SBP, DBP
Ruggenenti et al. [21]	RCT	Type 2 diabetics	25% CR	6 months	SBP, DBP, MAP
Hong et al. [24]	Obs	Some with MetS	500–800 kcal/day	12 weeks	SBP, DBP
Most et al. [26]	Obs	Normal weight	25% CR	24 months	SBP, DBP
Case et al. [31]	Obs	Obese, some with MetS	600–800 kcal/day	Various	SBP, DBP
Beleslin et al. [32]	Obs	Morbid obesity	500–800 kcal/day	3 weeks	SBP, DBP
Goldhamer et al. [34]	Obs	Pre-hypertensive	Water fasting	2 weeks	SBP, DBP
Goldhamer et al. [35]	Obs	Hypertensive	Water fasting	10–11 days	SBP, DBP
Ghachem et al. [36]	Obs	Obese, post-menopausal females	CR, high carbohydrate, low fat	6 months	No change
Hoddy et al. [40]	Obs	Obese	ADF 25% CR	8 weeks	No change
Harvie et al. [45]	Obs	Overweight or obese	25% CR 7 days/week or VLCD 2 days/week	6 months	SBP, DBP in both groups

RCT = randomised controlled trial; Obs = observational study; MetS = metabolic syndrome; CR = caloric restriction; ADF = alternate day fasting; VLCD = very low calorie diet; SBP = systolic blood pressure; DBP = diastolic blood pressure; MAP = mean arterial pressure.

**Table 2:** Summary of studies showing the effect of fasting or caloric restriction on lowering heart rate in >20 human subjects.

Study Author and Reference	Type of Study	Subjects	CR or No. of Calories	Duration of Study	Effect on HR and HRV
Stein et al. [8]	Obs	Healthy	30% CR	3–15 years	HR lower; HRV higher
Ruggenenti et al. [21]	RCT	Type 2 diabetics	25% CR	6 months	HR lower
Hoddy et al. [40]	Obs	Obese	ADF with 75% CR	8 weeks	HR no change
Meyer et al. [46]	Obs	Healthy	1672 kcal/day vs. 2445 kcal/day	Mean 6.5 years	HR no difference
Trepanowski et al. [52]	RCT	Obese	ADF with 75% CR, 25% CR or control	6 months	HR no change

RCT = randomised controlled trial; Obs = observational study; CR = caloric restriction; ADF = alternate day fasting; HR = heart rate; HRV = heart rate variability.

**Table 3:** Summary of studies showing the effect of fasting or caloric restriction on endothelium-dependent flow-mediated dilatation in >20 human subjects.

Study Author and Reference	Type of Study	Subjects	CR or No. of Calories	Duration of Study	Effect on Flow-Mediated Dilatation
Raitakari et al. [20]	Obs	Obese	580 kcal/day	6 weeks	Improved
Sasaki et al. [33]	Obs	Obese, hypertensive	800 kcal/day	2 weeks	Improved
Khoo et al. [67]	Obs	Diabetic, obese males	1000 kcal/day	8 weeks	Improved
Clifton et al. [68]	Obs	Overweight	Low fat/high carbohydrate CR	3 months	No change
Volek et al. [69]	Obs	Overweight	1500 kcal/day, low fat vs. low carbohydrate CR	12 weeks	Improved in low carbohydrate CR
